# The Continuity of the Germ Plasm as Foundation of Heredity

**Published:** 1917-05

**Authors:** S. R. Klein

**Affiliations:** Director of Norwich Research Laboratory, Formerly Professor Fordham University Medical School, Norwich, Conn.


### Journal-Record of Medicine

Successor to Atlanta Medical and Surgical Journal, Established 1855
and Southern Medical Record, Established 1870

**OWNED BY THE ATLANTA MEDICAL JOURNAL COMPANY**

PUBLISHED MONTHLY

*Official Organ Fulton County Medical Society, State Examing Board, Atlanta, Birmingham and Atlantic Railroad Surgeon's Association, Chattahoochee Valley Medical and Surgical Association, Etc.*

A. W. STIRLING, M. D., C. M., D. P. H.; J. S. HURT, B. Ph., M.D.
GEO. M. NILES, M. D., W. J. LOVE, M. D., (Ala.); Associate Editors

E. W. ALLEN, Business Manager

**COLLABORATORS**

W. F. WESTMORELAND, M. D., General Surgery
F. W. McRAE, M. D., Abdominal Surgery
ALLEN H. BUNCE, Medical Societies
E. B. BLOCK, M. D., Diseases of the Nervous System
MICHAEL HOKE, M. D., Orthopedic Surgery
CYRUS W. STRICKLER, M. D., Legal Medicine and Medical Legislation
E. C. DAVIS, A. B., M. D., Obstetrics
E. G. JONES, A. B., M. D., Gynecology
ALLEN H. BUNCE, M. D., Pathology and Bacteriology
R. T. DORSEY, JR., B. S., M. D., Medicine
L. M. GAINES, A. B., M. D., Internal Medicine
GEO. C. MIZELL, M. D., Diseases of the Stomach and Intestines
L. B. CLARKE, M. D., Pediatrics
EDGAR PAULLIN, M. D., Opsonic Medicine
THEODORE TOEPEL, M. D., Mechano Therapy
E. G. BALLENGER, M. D., Diseases of the Genito-Urinary Organs

VOL. LXIV. ATLANTA, GA, MAY, 1917. NO. 2

### THE CONTINUITY OF THE GERM PLASM AS FOUNDATION OF HEREDITY.

By S. R. Klein, M. D., Ph. D., M. A., Director of Norwich Research Laboratory, Formerly Professor Fordham University Medical School, Norwich, Conn.

1916 means the 50th anniversary of the birth of Biology. In 1876 Weisman brought forward his theory of heredity. This was a step in the doctrine of evolution. 20 years previously were already discovered minute bodies in animals that had died of anthrax by Pollender and Devaine, but the divisions of the parent cell was reall ydiscovered by Weisman. Pollender and Devaine called the discovered bodies bacteria, but did not yet consider them the cause of the disease. Nobody dreamed of asking why wounds gangrened, grapes moulded, wine soured, etc. But in 1857 Pasteur took up the study of fermentaentation and showed conclusively that its cause is the presence and growth of a micro-organism. Devaine in 1863 reinvestigated anthrax from this point of view and showed present in the blood of animals that had died of the disease.

In 1870 Koch—by that time a simple physician of Wollstein took up the subject and invented his method of pure cultivation of the bacteria by cultivating them in a thin layer of jelly, between glass plates, picking out species, and thus recultivating and selecting until the product was absolutely of one kind.

Pasteur and Cohn, by a series of the most careful experiments, conducted independently, gave the final blows to the spontaneous generation theory—the early seventies.

Pasteur began applying his method of inoculation about 1876· The first successful experiment was an animal inoculated with anthrax. The uninoculated died; the protected suffered no harm. Inoculation for chicken cholera was also successful. These first achievements were followed by a long series of famous discoveries, especially those of the germ of tuberculosis (Koch 1882); Typhoid fever (Eberth 1880); Diphtheria (Klebs Loffler 1883); Cholera (Koch 1884); Lockjaw (Nicholaier 1884); The grip (Canon 1892); Pneumonia (Frankel 1886); Pasteur's treatment for hydrophobia and Behring's Antitoxin give great hopes for the ultimate success of inoculation. The discoveries of these ''special'' bacteria—as they are—will probably solve the great problem and questions of the theory of heredity—as the bacillus of anthrax causes—anthrax only—the pneumococcus—pneumonia only and nothing else. And, consequently, the productive single cells are responsible for the production and reproduction of tissues and bodies consisting of the same cells—and these same cells are containing and producing always the same bacteria, the producers of disease. The lung cells, for instance the tuberculosis bacillus, the intestinal cells, the typhoid bacillus, etc., and when we put up the question, "How is it that such a single cell can reproduce the tout ensemble of the parent with all the faithfulness of a portrait?" It is not so hard to answer, as it seemed to solve the question 50 years ago, previously to the discoveries of the bacteria system.

Haecker's "Perigenesis of the Plastitude" and Darwin's "Pangenesis" can not be regarded as the O. K. answer, as Haeckel and Darwin did not know of the continuity of the germ plasm. Pangenesis is a modern revival of the oldest theory of heredity, that of Democritus, according to which the sperm is secreted from all parts of the body of both sexes during copulasion and is animated by a bodily force; according to this theory also, the sperm from each part of the body reproduced the same part.

If, according to the physiological and morphological ideas of the day, it is impossible to imagine that gemmules produced by each cell of the organism are at all times to be found in all parts of the body, and furthermore that these gemmules are collected in the sexual cells, which are then able to again reproduce in a certain order each separate cells of the organism, so that each sexual cells is capable of developing into the likeness of the parent body; if all this is inconceivable, we must inquire for some other way in which we can arrive at a foundation for the true understanding of heredity. The continuity of the germ plasm is founded upon the idea that heredity is brought about by the transference from one generation to another of a substance with a definitive chemical, and above all, molecular constitution. The germ plasm possesses a highly complex structure, conferring upon it the power of developing into a complex organism.

It is a certainty that a part of the specific germ plasm contained in the parent egg cell is not used up in the construction of the body of the offspring, but is reserved unchanged for the formation of the germ cells of the following generation. The specific qualities of organisms are based upon nuclei, mocecular stimuli proceed from the nucleus into the surrounding cytoplasm; stimuli which control the phenomena of assimilation in the cell and give to the growth of the cytoplasm a certain character peculiar to the species·

The opinion that the nucleus impresses its specific character upon the cell, has received important confirmation in the experiments upon the regeneration of Infusoria, conducted by Professor Nussbaum, of Boun and Professor Gouber of Fresburg, Germany.

Nussbaum's statement that an artifically separated portion of a Paramaecium, which does not contain any nuclear substance, immediately dies, must not be accepted of general application, for Professor Gruber has kept similar fragments of other Infusoria alive for several days. Moreover, Gruber had previously shown that individual protazoa occur, which live in a normal manner, and are yet without a nucleus, although this structure is present in other individuals of the same species. But the meaning of the nucleus is made clear by the fact published by Gruber, that such artifically separated fragments of Infusoria are incapable of regeneration, while on the other hand those fragments which contain nuclei always regenerate. And now here is the point! Those probozoa, which do not contain nuclei, will take a pathological form, as for instance the spirochaete of syphilis and although they cannot regenerate to a physiological form, but can live in the human tissues, producing luetic conditions, a form of pathological (syphilitic) chains, not known ever new to us. But those probozoa, without nuclei can stay in the human tissues from generation to generation, producing the hereditary syphilis—and they consist of plasma only.

